# The thrombomodulin analog Solulin promotes reperfusion and reduces infarct volume in a thrombotic stroke model

**DOI:** 10.1111/j.1538-7836.2011.04269.x

**Published:** 2011-06-06

**Authors:** E J SU, M GEYER, M WAHL, K MANN, D GINSBURG, H BROHMANN, K U PETERSEN, D A LAWRENCE

**Affiliations:** 1 Department of Internal Medicine, University of Michigan Medical SchoolAnn Arbor, MI, USA; 2 Howard Hughes Medical Institute, University of Michigan Medical SchoolAnn Arbor, MI, USA; 3 PAION Deutschland GmbHAachen, Germany

**Keywords:** anticoagulants, cerebral-ischemia, hemorrhage, stroke, thrombosis

## Abstract

*See also* Andreou AP, Crawley JTB. Thrombomodulin analogues for the treatment of ischemic stroke. This issue, pp 1171–3.

**Summary:**

*Background:* Currently there is no approved anticoagulant for treating acute stroke. This is largely because of concern for hemorrhagic complications, and suggests a critical need for safer anticoagulants. Solulin is a soluble analog of the endothelial cell receptor thrombomodulin, able to bind free thrombin and convert it to an activator of the anticoagulant, protein C. *Objective:* Solulin was tested for its ability to inhibit middle cerebral artery occlusion (MCAO) induced by photothrombosis, and to restore MCA patency after establishment of stable occlusion. *Methods:* Cerebral blood flow (CBF) was monitored by laser Doppler for 1.5 h after occlusion and again 72 h later. *Results:* Solulin treatment 30 min before thrombosis resulted in an approximately 50% increase in time to form a stable occlusion. When administered 30 or 60 min after MCAO, Solulin significantly improved CBF within 90 min of treatment. In contrast, none of the vehicle-treated mice showed restoration of CBF in the first 90 min and only 17% did so by 72 h. Solulin treatment was associated with a significant reduction in infarct volume, and was well tolerated with no overt hemorrhage observed in any treatment group. Mechanistic studies in mice homozygous for the factor (F)V Leiden mutation, suggest that Solulin’s efficacy derives primarily from the anticoagulant activity of the thrombin–Solulin complex and not from direct anti-inflammatory or neuroprotective effects of Solulin or activated protein C. *Conclusions:* Our data indicate that Solulin is a safe and effective anticoagulant that is able to antagonize active thrombosis in acute ischemic stroke, and to reduce infarct volume.

## Introduction

Stroke is the leading cause of morbidity and the third leading cause of mortality in the United States [1]. Approximately 80% of acute strokes are ischemic with the rest being hemorrhagic. Outcomes for hemorrhagic strokes are generally worse than for ischemic strokes and hemorrhagic conversion of an ischemic stroke markedly increases stroke severity [2]. Thus, although the nature of ischemic stroke suggests the need for anticoagulant intervention, there are currently no approved anticoagulants for treating acute stroke. Similarly, low-dose aspirin is the only anti-platelet drug currently recommended, and only within the first 24–48 h in patients with mild stroke [3], and all anticoagulant/platelet therapy is contraindicated within 24 h of thrombolysis with tissue type plasminogen activator (tPA) [4]. Many clinical studies have investigated anticoagulants in ischemic stroke, with the rationale that treatment would prevent ongoing thrombosis, maintain collateral circulation, halt recurrent stroke and reduce neurological deficits. In spite of these studies, the effectiveness of anticoagulants is still controversial, with a recent study suggesting that early anticoagulation can provide benefit, but at the risk of increased symptomatic brain hemorrhages [5]. Likewise, a meta-analysis of data from eight separate clinical trials, involving more than 22 000 participants, found that while early anticoagulation did reduce the incidence of recurrent ischemic stroke, this benefit was offset by an increase in symptomatic intracranial hemorrhages, and that overall there was no evidence that anticoagulation reduced mortality or dependence [6]. Thus, these studies suggest that a safer anticoagulant therapy could provide significant benefit in ischemic stroke patients.

An important innate system for controlling thrombosis is activation of the serine protease Protein C, to activated protein C (APC), a potent anticoagulant that inactivates coagulation cofactors Va and VIIIa [7,8]. APC is generated by thrombin; however, this process is extremely inefficient in the absence of the cofactor, thrombomodulin. Thrombomodulin is a receptor present on the luminal surface of intact endothelium that binds thrombin with high affinity (*K*_d_ = 0.5–1 nm) and increases its activation of protein C by more than 20 000-fold, switching thrombin from a procoagulant to an anticoagulant enzyme [7,9]. Thrombomodulin-bound thrombin can also activate the thrombin-activatable fibrinolysis inhibitor (TAFI) [10,11]. Thus, thrombomodulin naturally limits thrombosis at sites where the endothelium is intact, and through TAFI it can also moderate thrombolysis. The dependency on active free thrombin makes thrombomodulin an attractive candidate as an anticoagulant as it will exert anticoagulant activity only after the initiation phase of thrombin generation and hence will not impact formation of the primary hemostatic plug.

Solulin is a recombinant soluble analog of human thrombomodulin. It is a single-chain glycoprotein consisting of the extracellular domains of thrombomodulin modified by a few specific mutations and deletions that distinguish Solulin from similar products such as thrombomodulin alfa (ART-123) (Data S1). Otherwise, Solulin shares with thrombomodulin its essential mechanisms of action, including binding active thrombin and activation of protein C and TAFI [11]. The requirement for active thrombin restricts Solulin’s activity to sites with increased soluble thrombin, which may improve its safety profile and reduce the risk of hemorrhagic complications in stroke patients. The anticoagulant properties of Solulin have been verified in previous studies in rodent models [12] (data not shown) and are similar to those reported for thrombomodulin alfa [13].

In the present study, Solulin was tested for its ability to prevent thrombosis in the middle cerebral artery (MCA), restore MCA patency after MCAO, improve outcomes as measured by stroke lesion volume, and to do so without causing intracerebral hemorrhage. Finally, the mechanism of Solulin’s action was assessed by examining its efficacy in mice homozygous for the factor (F)V Leiden mutation, which makes its carriers resistant to the anticoagulant activity of APC [8,14].

## Methods

### Ischemic stroke model

Male C57Bl/6J and FV Leiden mice [14] (10 weeks) were anesthetized with chloral hydrate (450 mg kg^−1^) and placed securely under a dissecting microscope. The left MCA was exposed as described [15], and a laser Doppler flow probe (Type N: Transonic Systems, Ithaca, NY, USA) was placed on the surface of the cerebral cortex located 1.5 mm dorsal median from the bifurcation of MCA. The probe was connected to a flowmeter (Transonic model BLF21) and relative tissue perfusion units (TPU) was recorded with a continuous data acquisition program (windaq, dataq, Akron, OH, USA). Rose Bengal (RB) (Fisher) was diluted to 10 mg mL^−1^ in Lactate Ringer’s and then injected into the tail vein (50 mg kg^−1^). A 3.5-mW green light laser (540 nm; Melles Griot, Albuquerque, NM, USA) was directed at the MCA from a distance of 6 cm, and the TPU of the cerebral cortex was recorded. Stable occlusion was achieved when the TPU dropped to < 20% of pre-occlusion levels and did not rebound within 10 min after Laser withdrawal. All animal experiments were approved by the Institutional Animal Care and Use Committee of Unit for Laboratory Animal Medicine at University of Michigan, and followed the STAIR recommendations for preclinical studies (Data S1).

### Solulin delivery and cerebral blood flow tracing

Solulin was administered via a 26-G Abbocath®-T vascular catheter (Hospira, Lake Forest, IL, USA) inserted into the tail vein and connected to a Genie Plus syringe pump (Kent Scientific, Torrington, CT, USA). Mice received either 200 μL of Lactate Ringer’s (controls) or 200 μL of Solulin (1, 3 or 10 mg kg^−1^) either 30 min before RB injection, or 30 or 60 min after MCAO.

All cerebral blood flow (CBF) tracings were started 10 min before RB injection and the average CBF over this time was considered 100% and used to normalize CBF. Time zero was set at RB injection. Seventy-two hours after MCAO, animals were re-anesthetized with chloral hydrate (450 mg kg^−1^) and the surgical site was re-exposed and the Doppler flow probe was re-attached to the same location as before to obtain 72-h CBF data.

### Stroke volume

The assessment of stroke volume was performed essentially as described [15]. Briefly, brains were removed and cut into 2-mm-thick coronal sections and stained with 4% 2,3,5-triphenyltetrazolium chloride (TTC) in phosphate-buffered saline (PBS) for 20 min at 37 °C, and then fixed in 4% paraformaldehyde solution for 10 m. The sections were analyzed with nih image j using the following formula [16]:

*V*_%stroke_ = ∑(Areas of lesion)/∑(Areas of ipsilateral hemisphere) × 100,

where *V*_%stroke_ is stroke volume calculated as percent of the ipsilateral hemisphere.

### Hemoglobin assay

Twenty-four hours after MCAO, brains were removed, separated into hemispheres ipsilateral and contralateral to the MCAO, and each hemisphere was homogenized in 475 μL of PBS on ice. Approximately 25 μL of 10% Triton X-100 was added to a final concentration 0.5%. After mixing, samples were incubated at 23 °C for 5 min and then centrifuged at 25 000 *g* at 4 °C for 30 m. The absorbance of 50 μL of the supernatant was read at 410 nm and the hemoglobin quantified relative to a purified hemoglobin standard (Sigma-Aldrich, St. Louis, MO, USA) as described [15].

### Immunohistochemistry

Paraffin-embedded sections (5 μm) from vehicle- and Solulin-treated animals euthanized 72 h after MCAO were examined using the Apoptag kit (Oncor, Gaithersburg, MD, USA) according to the manufacturer’s instructions. The slides were developed with peroxidase substrate diaminobenzidinetetrahydrochloride for 5 min (Sigma-Aldrich), washed in Milli-Q deionized H_2_O for 5 min and counterstained with 0.5% methyl green for 10 min. To quantify cells with apoptotic bodies, an area surrounding the ischemic core extending from the cerebral cortex to the most anterior (septal) part of the hippocampus was imaged in vehicle and Solulin-treated animals. Three random fields were chosen and TUNEL positive cells were quantified under a 40 × objective [17].

### Statistical analysis

Data were analyzed using Student′s *t*-test (Excel; Microsoft, Redmond, WA, USA) and anova with Bonferroni’s Post Test (Prism; graphpad, La Jolla, CA, USA), *P* ≤ 0.05 was considered significant.

## Results

### Solulin extends time to occlusion in a photothrombotic model

**Figure 1:**
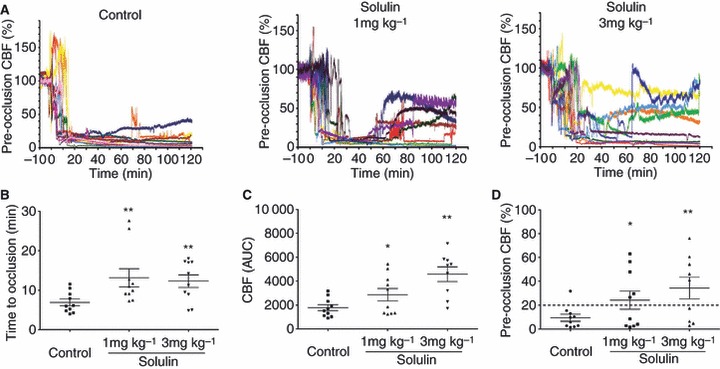
Solulin inhibits ongoing thrombosis in the middle cerebral artery (MCA). Time zero was set at Rose Bengal (RB) injection. Cerebral blood flow (CBF) tracings were started 10 min before RB injection and the average CBF from −10 to 0 was considered 100% and used to normalize all CBF measurements. (A) Individual CBF tracing after treatment with either control or Solulin (1 or 3 mg kg^−1^) and (B) analysis of time needed for stable occlusion after RB injection. Time to occlusion was measured from time of RB injection (30 min after injection of Solulin) to the time a stable occlusion was formed. (C) Analysis of relative vascular patency in MCA. Area under the curve (AUC) of each CBF tracing from RB injection to 120 min post injection was integrated. (D) Analysis of reperfusion. CBF tracings from 85 to 90 min after RB injection were normalized against pre-occlusion CBF to show individual data and averages (horizontal lines). The dashed line shows 20% of pre-occlusion CBF and acts as an arbitrary cutoff point for signs of reperfusion. Each group has *n* = 10 except the 3 mg kg^−1^ group, which has *n* = 9 and errors represent standard error of the mean (SEM) *P < 0.05 vs. control, and **P < 0.01.

We hypothesized that Solulin might be an effective and safe alternative to existing anticoagulants in the setting of thrombotic stroke. To determine Solulin’s capacity to prevent thrombosis, we compared intravenous bolus injections of 1 or 3 mg kg^−1^ of Solulin in a photothrombotic model of MCAO. Solulin was administered, and 30 min later thrombosis was initiated by the injection of the photoactive dye, RB and its local activation with a 540 nm laser. Laser Doppler flow measurements were initiated 10 min before RB injection to obtain baseline pre-occlusion CBF values and monitoring was continued for at least 120 min. These data demonstrated that Solulin treatment significantly increased the time to stable occlusion of the MCA from an average of 7.5 min in control animals to 13 or 12.5 min in the 1 or 3 mg kg^−1^ Solulin-treated animals, respectively (Fig. 1A,B). Furthermore, Solulin promoted reperfusion, as demonstrated by increased CBF at later times in both groups of Solulin-treated mice compared with controls (Fig. 1A). To quantify the relative reperfusion, the area under the CBF curves (AUC) was integrated and this analysis showed a significant increase with both 1 and 3 mg kg^−1^ Solulin treatment (Fig. 1C). Reperfusion was also analyzed by determining the average CBF values 90 min after RB injection (Fig. 1D). The dashed line indicates an arbitrary cutoff for animals considered to show signs of reperfusion (CBF > 20% of pre-occlusion levels). Only one out of 10 control mice was above this cutoff, whereas five out of 10 animals showed improved reperfusion both in the 1 and 3 mg kg^−1^ Solulin-treated groups, and this pattern persisted 72 h later (not shown). Moreover, both Solulin-treated groups showed statistically more reperfusion than the control animals when all animals were analyzed regardless of the cutoff line (Fig. 1D). This suggested that Solulin reduced the thrombotic response, and in doing so may have tipped the balance towards endogenous thrombolysis.

### Solulin increases reperfusion after MCAO

**Figure 2:**
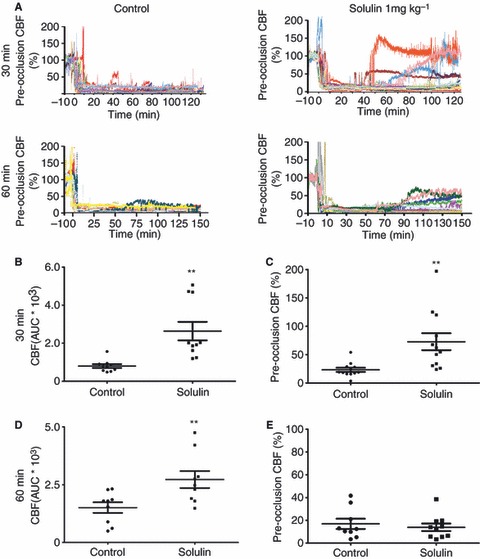
Solulin partially restores middle cerebral artery (MCA) patency after establishment of stable occlusion. (A) Cerebral blood flow (CBF) for mice treated with vehicle or 1 mg kg^−1^ of Solulin at either 30 or 60 min post occlusion. (B, D) Quantitative analysis of relative vascular patency in MCA from the CBF tracings in panel A, with injection 30 min (B) or 60 min (D) after occlusion. The area under the curve (AUC) of the CBF tracings was integrated for 90 min from the time of Solulin or vehicle injection. (C and E) Analysis of relative vascular patency in MCA 72 h post occlusion. CBF was determined over a 5-min period and normalized to pre-occlusion CBF. Each group *n* = 10 and errors represent standard error of the mean (SEM) **P < 0.01.

To test whether Solulin was effective after a stable occlusion of the MCA had already formed, Solulin, 1 mg kg^−1^, was administered either 30 or 60 min after MCAO. Figure 2A shows continuous Laser Doppler monitoring of CBF for up to 2.5 h after RB injection. Occlusion of the MCA began shortly after RB injection and was stabilized between 5 and 10 min after injection in all mice. In vehicle-injected mice the occlusion remained stable for the duration of the monitoring in all mice. In contrast, CBF tracings of mice treated with Solulin 30 or 60 min after MCAO showed that after Solulin delivery there was a partial restoration of CBF, beginning approximately 20 min after the initiation of Solulin administration. The extent of this reperfusion induced by Solulin treatment was calculated by integrating the area under the CBF curves (AUC), and these data demonstrated a statistically significant increase in CBF for both Solulin-treatment groups (Fig. 2B,D). When CBF was analyzed again 72 h after the initial occlusion, an average of approximately 73% of the pre-occlusion CBF value was observed in the 30-min Solulin-treated group (Fig. 2C). However, reperfusion at 72 h did not persist in the 60-min Solulin-treated group (Fig. 2E). These data demonstrated that Solulin markedly increased vascular reperfusion in the MCA within the first 2 h after MCAO. This benefit in reperfusion was sustained for up to 3 days if Solulin was initiated 30 min after MCAO (Fig. 2C), but was transient when Solulin administration was delayed until 60 min after MCAO (Fig. 2E).

### Solulin reduces infarct volume in wild-type mice

**Figure 3:**
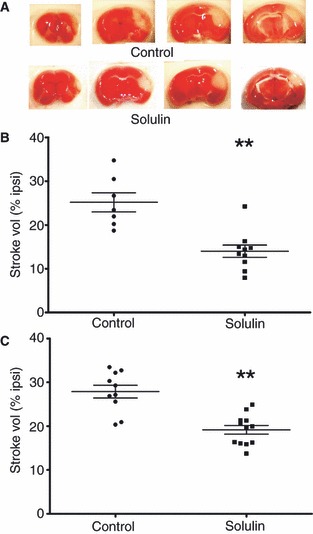
Solulin reduces infarct volume in wild-type mice. (A) Representative images of 2,3,5-triphenyltetrazolium chloride (TTC)-stained 2-mm-thick coronal sections with 1 mg kg^−1^ Solulin treatment 60 m after middle cerebral artery occlusion (MCAO). (B) Quantification of infarct size 72 h after MCAO in wild-type mice treated with either Solulin or vehicle 30 min after MCAO (*n* = 6 for control and *n* = 10 for Solulin). (C) Quantification of infarct size 72 h after MCAO in wild-type mice treated with either Solulin or vehicle 60 min after MCAO [*n* = 10 for control and *n* = 12 for Solulin. Errors represent standard error of the mean (SEM)], **P < 0.01.

As increasing early reperfusion during ischemic stroke may impact infarct expansion and improve stroke outcome, we tested whether Solulin treatment also mitigates brain damage. For this analysis stroke lesion volumes were compared in wild-type mice treated with Solulin (1 mg kg^−1^) or vehicle either 30 or 60 min after MCAO. Animals treated with Solulin showed a significant 48.8% or 30.6% reduction in stroke volume compared with vehicle-treated mice when Solulin was administered 30 (Fig 3B) or 60 min (Fig. 3A–C) after MCAO, respectively. These data suggested that Solulin produced marked beneficial effects after stroke.

### Solulin does not increase hemorrhagic transformation after MCAO

**Figure 4:**
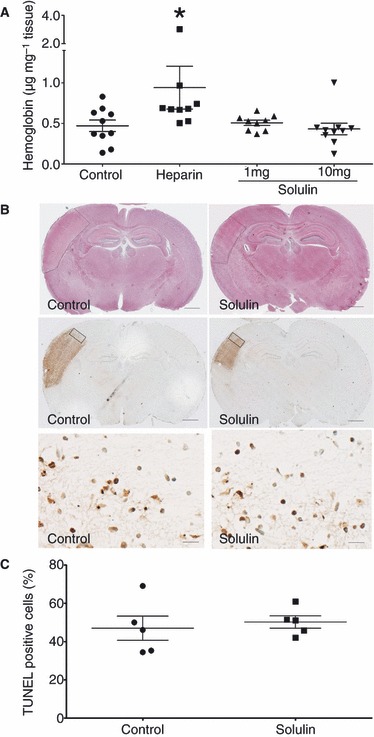
Quantification of hemoglobin and TUNEL positive cells in brain tissue after middle cerebral artery occlusion (MCAO). (A) Quantification of cerebral hemoglobin 24 h after MCAO in wild-type mice treated with either Solulin (1 or 10 mg kg^−1^), heparin (100-U kg^−1^) or vehicle [*n* = 10 per group and errors represent standard error of the mean (SEM)]. Treatments were given intravenously 60 min after MCAO. *P < 0.05 vs. control. (B) Representative coronal sections, stained with hematoxylin and eosin stain (H&E) (top panels, outlined areas indicate infarct region) and TUNEL (middle panels) 72 h after MCAO. High-resolution images of TUNEL staining from a field in the penumbra are shown in the bottom panels. Scale bars in the top and middle are 1 mm, and 50 μm in the bottom. (C) Quantification of TUNEL positive cells in the penumbra region (rectangular boxes in B middle panels) 72 h after MCAO.

As one of the primary reasons that anticoagulants are not used in ischemic stroke is the potential risk of hemorrhagic complications, we examined whether Solulin treatment increased hemorrhage during cerebral ischemia. Vehicle or Solulin (1 or 10 mg kg^−1^) was administered 60 min post-MCAO. Twenty-four hours after MCAO, brain tissues were perfused with PBS and processed for hemoglobin measurement. Figure 4A shows that there is no significant difference in the average hemoglobin content in the brain tissue of the vehicle-treated group compared with the Solulin-treated groups, even when 10 times the efficacious dose of Solulin was used (10 mg kg^−1^). Furthermore, neither macroscopic nor microscopic examination of the infarct tissue showed overt signs of hemorrhage in Solulin-treated mice (not shown). These data suggest that Solulin does not increase the risk of hemorrhagic complications when given after MCAO. In contrast, heparin-treated animals showed significantly more hemoglobin in the brain after stroke, compared with either control- or Solulin-treated mice (Fig. 4A). This is consistent with previous reports in mice that heparin treatment after stroke can exacerbate intracerebral hemorrhage [16,18], and in human stroke patients where heparin treatment is associated with an increased incidence of hemorrhagic transformation [5].

### The effect of Solulin treatment on cell death in the penumbra after MCAO

Analysis of neuronal cell death in the penumbra of the infarct was performed using immunohistochemical TUNEL staining. Quantification of TUNEL-positive cells in the region shown in the box in Fig. 4B demonstrated that there was no difference in the extent of neuronal death in the penumbra between the Solulin-treated and untreated groups (Fig. 4C). This suggests that Solulin was not acting as a direct neuroprotectant but reduced overall infarct size by acting as an anticoagulant and improving CBF.

### Solulin’s protective effect absent in FV Leiden mice

**Figure 5:**
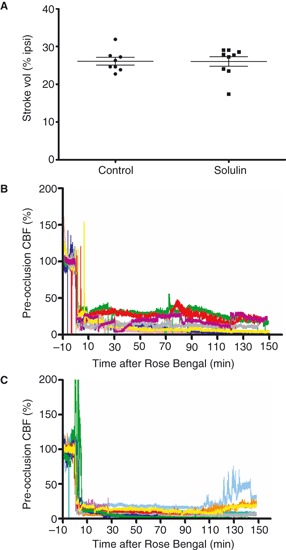
Solulin is not protective in factor (F)V Leiden mice. (A) Quantification of infarct size 72 h after middle cerebral artery occlusion (MCAO) in FV Leiden mice treated with either Solulin (1 mg kg^−1^) or vehicle [*n* = 8 for control and *n* = 9 for Solulin; errors represent standard error of mean (SEM)]. Solulin was given via intravenously 60 min after MCAO. In panels B (vehicle) and C (Solulin), Time zero was set at Rose Bengal (RB) injection. All cerebral blood flow (CBF) tracings were started 10 min before RB injection and the average CBF over this time was used to normalize other CBF measurements.

To further examine whether Solulin’s protective effect after MCAO was because of its anticoagulant activity or mediated by neuroprotective or anti-inflammatory effects described for both thrombomodulin/Solulin and APC, Solulin was administered to FV Leiden mice [14] 60 min after stroke induction, and stroke lesion volumes were evaluated 72 h after MCAO (Fig. 5A). These data demonstrate that unlike wild-type animals (Fig. 3A,B), FV Leiden mice treated with Solulin did not show any reductions in infarct size. This implies that the protection mediated by Solulin is directly related to its APC-mediated anticoagulant activity, rather than other effects of Solulin, or APC, as in FV Leiden mice Solulin should still bind thrombin and stimulate APC generation to the same extent as wild-type mice, but would differ in their specific resistance to the anticoagulant activity of APC. Consistent with this hypothesis, CBF tracings of FV Leiden mice confirmed that Solulin treatment did not produce a significant increase in reperfusion after MCAO (Fig. 5B,C). This suggests that Solulin’s protective effect is dependent on its anticoagulant activity, mediated by APC’s ability to inactivate FV.

## Discussion

Anticoagulants have been evaluated in several major clinical trials for efficacy in acute ischemic stroke, including warfarin, unfractionated heparin, low-molecular-weight heparin or heparinoids [5,6,19–21]. However, these studies have been inconclusive, failing to establish unequivocal beneficial effects on clinical outcome in either the short or long term. Instead, the anticoagulants studied have been invariably linked to a significant increase in intracranial bleeding, and so their use in acute ischemic stroke is contraindicated. Likewise, platelet inhibitors such as abciximab have been linked to increased intracranial hemorrhage and mortality, and failed to show efficacy, suggesting that this class of drugs does not offer a safe alternative [22]. Thus, stroke presents a unique set of challenges for the therapeutic use of anticoagulants.

Solulin, a soluble analog of human thrombomodulin that lacks the transmembrane and cytoplasmic domains of endogenous thrombomodulin, has been engineered to be resistant to oxidation and proteolysis, and to have a prolonged half-life in plasma (Data S1). Functionally, Solulin is equivalent to thrombomodulin in its primary extracellular mechanisms of action. Once thrombin generation occurs, Solulin binds to active thrombin and the thrombin–Solulin complex efficiently activates TAFI, and hence reduces thrombolytic activity [10,11]. However, at higher Solulin concentrations, activation of protein C predominates [23,24]. APC inactivates coagulation cofactors Va and VIIIa, curbing the thrombin burst that normally occurs in the propagation phase of coagulation, making Solulin an efficient anticoagulant [7,8]. APC also down-regulates TAFI activation and enhances thrombolytic activity [23,24]. Thus, thrombin’s ability to initiate clot formation prevails, while clot extension and maturation are inhibited. This mechanism is unlike those of existing anticoagulants which inactivate thrombin directly, and consequently Solulin may have a lower impact on hemostasis than seen with other anticoagulants.

The present study supports the hypothesis that Solulin treatment reduces stroke lesion volume through local activation of protein C, which shifts the coagulation/thrombolytic balance towards a reduced thrombus burden and increased cerebral perfusion. Pharmacokinetic data strongly suggest that the Solulin doses we employed should have markedly suppressed thrombin generation (Data S1). Intravenous injection of Solulin before the onset of thrombosis increased the time needed to form a stable occlusion in the MCA, and increased overall CBF, clearly demonstrating that Solulin is an effective anticoagulant. Solulin administration 30 min after stable MCAO also enhanced reperfusion, and reduced infarct volume by nearly 50%. This indicates that even after a stable thrombus has formed Solulin can still promote recanalization and improve outcome. In mice treated 60 min after MCAO, CBF in the territory immediately proximal to the MCAO was significantly improved only during the first 2-h observation period; however, infarct volume was still reduced by 30%. This suggests that while late treatment with Solulin may not be as effective as early delivery, it may still alter the coagulation/thrombolytic balance at sites distal to the MCAO, which may not be apparent in CBF measurements proximal to the MCAO, but may still improve reperfusion in the periphery of the ischemic zone enough to reduce infarct expansion.

To confirm the hypothesis that Solulin’s protective benefit after MCAO was mediated primarily by the generation of APC, we used a mutant mouse strain carrying the murine equivalent of the FV Leiden mutation, which makes FVa resistant to APC cleavage and inactivation. We hypothesized that if Solulin’s benefit in ischemic stroke was as a result of generation of APC, then FV Leiden mice should show no benefit from Solulin treatment. The present results supported this conjecture and demonstrated that the FV Leiden mice were not protected with regard to either stroke lesion volume or decreases in CBF compared with control animals. As these mice are directly resistant to the anticoagulant activity of APC then this suggests that the primary benefit of Solulin arises from its ability to promote APC-mediated anticoagulation through the inactivation of FVa. However, it is also possible that unrestrained thrombin generation in these mice could support direct activation of TAFI by thrombin, resulting in a hypothrombolyic state, as has been described with FV Leiden in both humans and mice [25,26]. Thus, it is not possible to definitively determine to what extent improvements in reperfusion and reductions in infarct volumes in wild-type mice were due directly to APC-mediated anticoagulant activity or indirectly to enhancement of endogenous thrombolytic activity. Nonetheless, both activities would be expected to reduce thrombus extension and stabilization, and to tip the balance from coagulation towards thrombolysis.

It is also possible that Solulin binding to thrombin could have direct anti-thrombin activity, preventing interaction with fibrinogen or other procoagulant substrates, or could provide direct neuroprotectection through its ability to reduce thrombin diffusion through a compromised blood–brain barrier, and the attendant thrombin neurotoxicity [27]. With regard to direct anti-thrombin activity, previous studies of thrombin-induced mortality in mice have shown that Solulin does not act by inhibiting active thrombin injected into mice, but instead acts by reducing the generation of additional thrombin [12]. With regard to direct neuroprotection, this too seems unlikely for two reasons: first, in the FV Leiden mice, Solulin should still bind thrombin and reduce its diffusion into the central nervous system (CNS), and yet the relative stroke volumes in Soluin-treated FV Leiden mice were indistinguishable from vehicle-treated FV Leiden mice. Second, thrombin neurotoxicity is generally associated with an increased loss of blood–brain barrier integrity and intracerebal hemorrhage [28,29], and no increase in overt intracerebal hemorrhage was noted in the FV Leiden mice.

APC has also been reported to be directly neuroprotective when infused immediately before or after MCAO, and this activity is independent of its anticoagulant activity [30,31]. However, our data do not support this mechanism for Solulin, as the FV Leiden mutation completely suspends Solulin’s efficacy even although Solulin should still promote generation of APC in these mice. The reason for this difference may be because of systemic effects associated with the direct administration of APC, or the timing of treatment relative to ischemia in the two cited studies, or more likely to differences in the local or systemic concentrations of APC.

In contrast to mice treated with heparin [18] (Fig. 4A), Solulin-treated animals showed no significant increases in brain hemorrhage after MCAO. This suggests that Solulin may have a better safety profile than existing anticoagulants. We speculate that this is as a result of the generation of anticoagulant activity only at sites where free active thrombin has reached a sufficient concentration. As a result, and unlike other anticogulants such as heparin [32], Solulin will not inhibit the initiation phase of coagulation, but only clot extension and maturation, and in the absence of active soluble thrombin, Solulin will be virtually inert. This unique characteristic of Solulin sets it apart from other anticoagulants and may increase its therapeutic potential.

It is also important to note that although Solulin’s primary mode of action appears to be through the activation of protein C, it does not appear to produce the same bleeding risk that the direct administration of APC does [33] (Data S1). The reduced susceptibility to bleeding associated with Solulin compared with APC is most likely because of markedly different concentration profiles of APC in the circulation during direct APC infusion compared with Solulin administration, as with Solulin, APC is generated only locally and incidental to thrombin generation. Finally, the safe use of Solulin in humans has been demonstrated in a recent phase I study, in which Solulin at single doses up to 30 mg, and multiple doses up to 10 mg, was able to reduce endogenous thrombin potential by a maximum of 98% without any signs of enhanced bleeding tendency [34].

In summary, our data provide *in vivo* evidence that Solulin facilitates recanalization and reduces stroke volume after MCAO, and supports the suggestion that Solulin may have utility as a novel anticoagulant, able to curb thrombin generation with a promising safety profile in cerebral ischemia. The efficacy of Solulin seems to be mediated through the local formation of APC, which inhibits further intravascular thrombin formation by inactivating coagulation cofactors Va and VIIIa and thus antagonizes occluding clot growth. Another significant consequence of thrombin down-regulation may lie in a suppression of TAFI activation, promoting effective clot lysis by endogenous tPA. These features enable robust but local antithrombotic activity without causing global impairment of hemostatic pathways. Future studies are needed to further explore the potential of Solulin for the treatment of ischemic stroke. These include its potential to prevent re-occlusion after thrombolysis or mechanical thrombectomy, and treatment at later times after MCAO as in the clinic it will be necessary to differentiate between hemorrhagic and ischemic stroke before Solulin can be administered.
