# Supplementary material for: The thrombomodulin analog Solulin promotes reperfusion and reduces infarct volume in a thrombotic stroke model
Source: J Thromb Haemost. 2011 Jun 6;9(6):1174–82. doi: 10.1111/j.1538-7836.2011.04269.x (PMC3111949; doi:10.1111/j.1538-7836.2011.04269.x)
Supplement: Data S1. — Supplement. [file jth0009-1174-SD1.pdf]

Supplement

Structure of Solulin

Solulin is a recombinant soluble analogue of human thrombomodulin (UniProt Database: http://www.uniprot.org/uniprot/P07204) produced from the genetically engineered Chinese Hamster Ovary (CHO) cell line DXB11 carrying a recombinant plasmid with the Solulin gene. Consisting of the extracellular domains of thrombomodulin, Solulin contains 487 amino acids in the mature secreted form. It is distinguished from other available forms of soluble thrombomodulin by several directed mutations: deletion of the first three amino acids of the amino terminus and the last 7 amino acids of the carboxyl terminus, and four single amino acid exchanges: $Met^{388} \rightarrow$ Leu, $Arg^{456} \rightarrow$ Gly, $His^{457} \rightarrow$ Gln, and $Ser^{474} \rightarrow$ Ala [1].

The deletion at the N terminus generates a strongly favored signal sequence cleavage site and abolishes the N terminal heterogeneity arising from two common cleavage sites in the wild type sequence. Similarly, removal of a portion of the C-terminal region of the polypeptide, which is not critical to biological function, leads to a polypeptide that terminates in a Pro-Pro sequence at amino acids 489-490, a sequence especially resistant to exocarboxypeptidase activity [2].

For the sake of enhanced protease stability and oxidation resistance, the trypsin cleavage site Arg^{456} - His^{457} has been replaced with Gly - Gln [3] and the Met^{388} with Leu [4]. A final point mutation is $Ser^{474} \rightarrow$ Ala, an exchange known to block chondroitin sulphate attachment to the serine/threonine-rich domain [5]. The efficacy of these point mutations has been verified for Solulin [1,3,6]. Experiments using soluble thrombomodulin (extracellular domains expressed in four different cell lines, no further modifications) indicated that the $Met^{388} \rightarrow$ Leu mutation conferred a substantial increase in the ability to activate protein C (1.4-2.2 fold in molecules lacking the chondroitin sulphate moiety like Solulin [7].

There are minor differences in domain definitions used for Solulin and the related molecule, thrombomodulin alfa (ART-123). Solulin consists of amino acids 4 to 490, of mature thrombomodulin. This represents a slightly truncated form of the extracellular domains of thrombomodulin, which have been defined as either residues 1-497 [8] or 1-496 [9]. In contrast, ART-123 is comprised of amino acids 1-498, and therefore contains 11 additional residues including either one or two amino acids from the transmembrane domain.

Relevant Pharmacodynamics and Pharmacokinetics

Solulin causes a concentration-dependent activation of TAFI [8,10] as well as of protein C [8,11-13]. In saline containing the minimum constituents only (thrombin and Solulin plus either PC or TAFI), half-maximal rates of activation were reached at ~ 5 nM for TAFI and ~ 20 nM for PC [8]. There are no published studies in normal plasma which show that Solulin shares with rabbit lung thrombomodulin the concentration-dependent transition from prolongation to shortening of clot lysis time due to a change from activation of TAFI to activation of PC [14,15]. However, preliminary data in haemophilic blood suggest very similar effects of Solulin (Data not shown).

Moreover, APC generation is predominant at higher concentrations of Solulin. In human volunteers, a plasma concentration of about 14 nM Solulin was associated

with suppression of the endogenous thrombin potential by approximately 90 % [16]. This effect strongly suggests that TAFI activation is hardly possible in this setting, neither by high concentrations of thrombin (which are averted by Solulin) nor by the Solulin/thrombin complex (whose formation will be attenuated by down-regulation of prothrombin activation). Pharmacokinetic studies in mice yield C_{max} ~ 100 nM, and elimination half life ~ 4,5 hr, after a single dose of 0.5 mg/kg of Solulin (Data not shown). These data are important for the present study since they indicate that Solulin concentrations produced at relevant times (0 – 60 post-administration) in mice by a dose of 1 mg/kg should clearly result in Solulin concentrations that are by far sufficient for efficient activation of PC.

Solulin and APC

The findings of intracerebal hemorrhage with APC are consistent with a general bleeding risk that is part of its clinical safety profile [17]. If proven in clinical practice, a lower bleeding propensity of Solulin could be explained by dissimilar concentration profiles of APC, being sizable throughout the circulation during direct infusion of APC but very low to nil with infusion of Solulin and only locally increasing upon incidental thrombin generation. Thus, APC will be included in the growing clot and readily antagonize clot growth, whereas Solulin first needs to react with thrombin to activate PC. This difference may also help to explain why APC, unlike solulin, prolongs the lag time of the thrombin burst [18-20], a gross measure of the initial clot appearance, which coincides with aPTT.

Ischemic stroke model and STAIR recommendations.

All animal experiments followed the STAIR recommendations for preclinical studies [21]. Sample sizes were calculated based on power analysis of previous studies with this model [22]. Animals were randomized to each treatment group, and treatments were given by the technician with no knowledge of the specific treatments, which were prepared beforehand by a second person. The only animals excluded from analysis were animals that died before the experiments were terminated. The overall mortality of the study was approximately 3% and was evenly spread between groups. All data analysis including stroke volumes, times to occlusion, CBF measurements, haemoglobin analysis, and TUNEL staining was performed by a blinded investigator with no knowledge of the treatment groups, and results were decoded by the senior investigator after analysis.

References

1 Weisel JW, Nagaswami C, Young TA, Light DR. The shape of thrombomodulin and interactions with thrombin as determined by electron microscopy. *J Biol Chem* 1996;**271**:31485-31490.

2 Light, D. R., Andrews, W. H., Clarke, J. H., Wydro, R. M., and Young, P. A. Protease-resistant thrombomodulin analog. *European Patent* 2004;EP1449849A2:1-62.

3 Light DR, Glaser CB, Betts M, Blasko E, Campbell E, Clarke JH, McCaman M, McLean K, Nagashima M, Parkinson JF, Rumennik G, Young T, Morser J. The interaction of thrombomodulin with Ca2+. *Eur J Biochem* 1999;**262**:522-533.

4 Glaser CB, Morser J, Clarke JH, Blasko E, McLean K, Kuhn I, Chang R-J, Lin J-H, Vilander L, Andrews WH, Light DR. Oxidation of a specific methionine in thrombomodulin by activated neutrophil products blocks cofactor activity. A potential rapid mechanism for modulation of coagulation. *J Clin Invest* 1992;**90**:2565-2573.

5 Lin JH, McLean K, Morser J, Young TA, Wydro RM, Andrews WH, Light DR. Modulation of glycosaminoglycan addition in naturally expressed and recombinant human thrombomodulin. *J Biol Chem* 1994;**269**:25021-25030.

6 Pawar SA, Fu Q, Petersen K-U, Weiler H, Fink LM, Hauer-Jensen M. Targeting the endothelial Thrombomodulin- Protein C pathway to mitigate radiation lethality. *56th Annual Meeting Radiation Research Society* 2010;73-74.

7 Clarke JH, Light DR, Blasko E, Parkinson JF, Nagashima M, McLean K, Vilander L, Andrews WH, Morser J, Glaser CB. The short loop between epidermal growth factor-like domains 4 and 5 is critical for human thrombomodulin function. *J Biol Chem* 1993;**268**:6309-6315.

8 Wang W, Nagashima M, Schneider M, Morser J, Nesheim M. Elements of the primary structure of thrombomodulin required for efficient thrombin-activable fibrinolysis inhibitor activation. *J Biol Chem* 2000;**275**:22942-22947.

9 Mohri M. ART-123: Recombinant human soluble thrombomodulin. *Cardiovascular Drug Reviews* 2000;**18**:312-325.

10 Bajzar L, Morser J, Nesheim M. TAFI, or plasma procarboxypeptidase B, couples the coagulation and fibrinolytic cascades through the thrombin-thrombomodulin complex. *J Biol Chem* 1996;**271**:16603-16608.

11 Hall SW, Nagashima M, Zhao L, Morser J, Leung LL. Thrombin interacts with thrombomodulin, protein C, and thrombin-activatable fibrinolysis inhibitor via specific and distinct domains. *J Biol Chem* 1999;**274**:25510-25516.

12 van 't Veer C, Golden NJ, Kalafatis M, Mann KG. Inhibitory mechanism of the protein C pathway on tissue factor-induced thrombin generation. Synergistic effect in combination with tissue factor pathway inhibitor. *J Biol Chem* 1997;**272**:7983-7994.

13 Oosting JD, Preissner KT, Derksen RH, de Groot PG. Autoantibodies directed against the epidermal growth factor-like domains of thrombomodulin inhibit protein C activation in vitro. *Br J Haematol* 1993;**85**:761-768.

14 Mosnier LO, Meijers JC, Bouma BN. Regulation of fibrinolysis in plasma by TAFI and protein C is dependent on the concentration of thrombomodulin. *Thromb Haemost* 2001;**85**:5-11.

15 Mosnier LO, Elisen MG, Bouma BN, Meijers JC. Protein C inhibitor regulates the thrombin-thrombomodulin complex in the up- and down regulation of TAFI activation. *Thromb Haemost* 2001;**86**:1057-1064.

16 van Iersel T, Stroissnig H, Giesen P, Wemer J, Wilhelm-Ogunbiyi K. Phase I study of Solulin, a novel recombinant soluble human thrombomodulin analogue. *Thromb Haemost* 2010;**105**: [Epub ahead of print].

17 Fumagalli R, Mignini MA. The safety profile of drotrecogin alfa (activated). *Crit Care* 2007;**11** Suppl 5:S6.

18 Dielis AW, Castoldi E, Spronk HM, Van OR, Hamulyak K, Ten CH, Rosing J. Coagulation factors and the protein C system as determinants of thrombin generation in a normal population. *J Thromb Haemost* 2008;**6**:125-131.

19 Hezard N, Bouaziz-Borgi L, Remy MG, Nguyen P. Utility of thrombin-generation assay in the screening of factor V G1691A (Leiden) and prothrombin G20210A mutations and protein S deficiency. *Clin Chem* 2006;**52**:665-670.

20 Regnault V, Beguin S, Wahl D, de ME, Coenraad HH, Lecompte T. Thrombinography shows acquired resistance to activated protein C in patients with lupus anticoagulants. *Thromb Haemost* 2003;**89**:208-212.

21 Fisher M, Feuerstein G, Howells DW, Hurn PD, Kent TA, Savitz SI, Lo EH. Update of the stroke therapy academic industry roundtable preclinical recommendations. *Stroke* 2009;**40**:2244-2250.

22 Su EJ, Fredriksson L, Geyer M, Folestad E, Cale J, Andrae J, Gao Y, Pietras K, Mann K, Yepes M, Strickland DK, Betsholtz C, Eriksson U, Lawrence DA. Activation of PDGF-CC by tissue plasminogen activator impairs blood-brain barrier integrity during ischemic stroke. *Nat Med* 2008;**14**:731-737.
